# Electrochemotherapy: from the drawing board into medical practice

**DOI:** 10.1186/1475-925X-13-29

**Published:** 2014-03-12

**Authors:** Damijan Miklavčič, Barbara Mali, Bor Kos, Richard Heller, Gregor Serša

**Affiliations:** 1 Faculty of electrical Engineering, Department of Biomedical Engineering, University of Ljubljana, Trzaska 25, Ljubljana SI-1000, Slovenia; 2 Frank Reidy Research Center for Bioelectrics, Old Dominion University, 4211 Monarch Way, Suite 300, Norfolk, Virginia 23508, USA; 3 Department of Experimental Oncology, Institute of Oncology Ljubljana, Zaloska 2, Ljubljana SI-1000, Slovenia

**Keywords:** Electroporation, Electrochemotherapy, Preclinical results, Clinical studies, Electrochemotherapy for skin and superficial tumors, Electrochemotherapy for visceral and deep-seated tumors

## Abstract

Electrochemotherapy is a local treatment of cancer employing electric pulses to improve transmembrane transfer of cytotoxic drugs. In this paper we discuss electrochemotherapy from the perspective of biomedical engineering and review the steps needed to move such a treatment from initial prototypes into clinical practice. In the paper also basic theory of electrochemotherapy and preclinical studies *in vitro* and *in vivo* are briefly reviewed. Following this we present a short review of recent clinical publications and discuss implementation of electrochemotherapy into standard of care for treatment of skin tumors, and use of electrochemotherapy for other targets such as head and neck cancer, deep-seated tumors in the liver and intestinal tract, and brain metastases. Electrodes used in these specific cases are presented with their typical voltage amplitudes used in electrochemotherapy. Finally, key points on what should be investigated in the future are presented and discussed.

## Introduction

Electrochemotherapy is a local treatment of cancer which combines the use of a medical device with pharmaceutical agents to achieve local tumor control in solid cancers. The procedure consists of applying short high-intensity pulsed electric fields to cells, in response to which, the plasma membrane’s permeability to various molecules transiently increases. This facilitates cellular uptake of cytotoxic agents, thus increasing their cytotoxicity [1-4]. The treatment is based on the phenomenon termed electroporation, which occurs, when externally delivered electric field induces a sufficiently large transmembrane voltage. Electroporation is, in addition to its use in electrochemotherapy, used also as non-viral gene delivery method to cells *in vitro* and *in vivo* – gene electrotransfer [5,6]. Furthermore, electroporation as sole modality can be used as tumor ablation in the form of irreversible electroporation, also referred to as non-thermal irreversible electroporation [7-14].

Electrochemotherapy uses electroporation to allow increased uptake of chemotherapeutic drugs into tumor cells [2,15]. Since the initial research *in vitro*, which was performed more than 20 years ago, electrochemotherapy has gone through extensive basic research on the mechanisms of its action, the required electric field, pulse parameters [16] and sequences for successful treatment, screening of appropriate drugs for use with electric pulses [17,18], *in vivo* studies on different animal models [19], as well as clinical trials in cutaneous and subcutaneous tumors [19,20] and deep-seated tumors [21-23]. The aim of this review is to give the reader a concise overview over the development of this biomedical engineering-based cancer treatment modality. Its foundation and discussion is critical to guide the development of electrochemotherapy, as adequate preclinical evidence for any kind of medical treatment requires significant time and financial resources. This present paper provides an overview of the current status of electrochemotherapy and sets the groundwork for discussion on further development and standardization of the technique in relation to other local and/or ablation treatment modalities.

This review is divided into several sections comprising the following topics:

• Overview of preclinical research and clinical data on use of electrochemotherapy in treatment of cutaneous and subcutaneous tumors. Implementation into standard of care, focusing on cutaneous and subcutaneous metastases.

• Other uses of electrochemotherapy, such as treatment of visceral and deep-seated tumors.

• The last section outlines the challenges for future studies which need to be addressed, taking into account important aspects of cancer treatment, such as improvement in patient’s quality of life and in time to progression or overall survival.

### Physical/theoretical background of electrochemotherapy

Electrochemotherapy relies on using hydrophilic drugs in combination with application of high-voltage electric pulses applied to tumors. The two drugs that have been used most often in electrochemotherapy are bleomycin (BLM), and cisplatin (*cis*-diamminedichloroplatinum (II) – CDDP). Both of these drugs are membrane low- or non-permeable, and their intracellular target is DNA [24]. The applied electric pulses are most commonly delivered in trains of eight 100-μs-long pulses. By using these electric pulses, the efficacy of the chemotherapeutic drugs is potentiated significantly. The potentiation is most expressed for BLM [2] and CDDP [25]. Potentiation of other drugs has also been studied [17,18] but proved to be much lower or insignificant (Table 1).

**Table 1:** **Summary of drugs tested for *in vitro *** **and *in vivo *** **potentiation in combination with electroporation pulses**

**Drug tested**	***In vitro *** **potentiation**	***In vivo *** **potentiation**
Bleomycin	Yes; 100–5000-fold [1,17,18,26-28]	Yes
Cisplatin	Yes; 1.8–12.2-fold [17,18,28,29]	Yes [28-30]
Calcium	Yes; more hundred-fold [31,32]	Yes [31]
Netropsin	Yes; 200-fold [1]	–
Carboplatin	Yes; 1.6–13-fold [17,18,27,29]	–
2-*N*-methyl-9-hydroxy-ellipticinium (NMHE)	Yes; 4-fold [1]	–
Vincristine	Yes; 1.3–3.4-fold [18]	–
ActiNomycin D	Yes; 2–3-fold [1]	–
Cytarabine	Yes; 2-fold [18]	–
Oxaliplatin	Yes [29]	–
Platinum (II) complex 3P-SK	Yes [29]	Yes [29]
Platinum (II) complex PtAMP	Yes [29]	–
Mitomycin C	Yes but low; 1.3–1.4-fold [18,33]	–
Vinblastine	Yes but low; 1.1–1.3-fold [18]	–
5-fluorouracil	No or low; 1.25-fold [18,26]	–
Paclitaxel	No or low; 1.3-fold [17,18]	–
Doxorubicin	No or low; 0.67–2-fold [17,18]	–
Nimustine hydrochloride (ACNU)	No [27]	–
Methotrexate	No [1]	–
9-OH-ellipticine	No [1]	–
Didemnin B	No [1]	–
Melphalan	No [1]	–
Mithramycin	No [1]	–
Taxotere	No [1]	–
DauNorubicin	No [17]	–
Adriamycin	No [32]	No [28]
Etoposide	No or ND [17,18,27]	–
Ancitabine	ND [18]	–
Gemcitabine	ND [18]	–

ND drug was tested but the potentiation could Not be determined due to methodological limitations.

– Not tested.

Upon the application of external electric pulses to cells (either in a culture medium, or in tissue), the electric field causes relocation of charges on the cell membrane. This causes the buildup of transmembrane voltage – termed induced transmembrane voltage, which is superimposed to the cells’ normal resting transmembrane voltage (or resting membrane potential, as it is termed in physiology literature). The increase in transmembrane voltage follows Schwann’s equation [34,35], and in areas, where transmembrane voltage exceeds a certain voltage, a sufficiently strong electric field through the membrane is established leading to pore formation which allows the passage of water, charged molecules, as well as larger molecules. Although these pores are too small and short-lived to be observed using conventional or electron microscopy, indirect evidence supporting their existence comes from simulations of lipid bilayers using molecular dynamics simulations [36-38].

Cellular membranes can remain permeable, for large and charged molecules, for minutes after the external electric field delivery ceases [4,39,40]. The mechanism of membrane “resealing” or repair requires active cellular mechanisms and therefore also energy [41]. A possible explanation of the comparatively long-term permeability of cell membranes is chemical alteration of membrane lipids [42-44]. In order to successfully, and reproducibly achieve cell membrane electroporation, which is the prerequisite for successful electrochemotherapy, appropriate pulse generators are needed. Initially, capacitor discharge pulse generators were used, but later square wave generators became the most prominent as they provide better reproducibility of pulses and control of electroporation [45].

### Preclinical research and electrochemotherapy mechanisms

Introduction of electrochemotherapy into clinical trials and nowadays into clinical practice is based on extensive preclinical data, on its effectiveness on different tumors and on solid evidence of its mechanisms of action. Several mechanisms of action have already been identified: increased membrane permeability and intracellular drug accumulation; vascular effects; and involvement of immune response.

The first and the predominant one is increased cellular uptake of BLM and CDDP, by exposure of cells or tumors to electric field. Increased cytotoxicity of BLM or CDDP was demonstrated *in vitro,* with several fold potentiation [2,18,25] (Table 1), as already mentioned above. The *in vitro* data were confirmed and elaborated *in vivo* on different animal tumor models [4,46-49]. Sufficient drug accumulation in cells is one of the most prominent underlying mechanisms responsible for effective electrochemotherapy [2,30,50]. Since the intracellular drug accumulation due to membrane permeabilization is a consequence of exposure of the cells to sufficiently high local electric field, therefore adequate electric field distribution in the tumors needs to be established [51,52]. All these preclinical data on the pharmacological and physical parameters needed for effective electrochemotherapy have translated into clinical use of electrochemotherapy. Currently, BLM is predominantly used in electrochemotherapy, based on higher potentiation of its cytotoxicity. However, the use of CDDP still remains to be fully explored. CDDP namely has the advantage of being effective already on its own, which however was not further explored after the initial clinical study [53]. Namely, widespread clinical use of CDDP in standard of care could be augmented by sensitizing specific tumors to CDDP by delivering electric pulses [54]. From the clinical perspective it is also helpful that there is another drug of choice, when BLM is contraindicated, or the allowed cumulative dose of BLM is reached [55].

Electrochemotherapy also has two distinct vascular effects. Since the exposure of tumors to electric fields predisposes stromal cells to drug uptake, electrochemotherapy also has an effect on endothelial cells of tumor vessels. This action leads to endothelial cell death (apoptosis) and consequently to abrogation of tumor blood flow. This first effect was named vascular disrupting effect of electrochemotherapy [56]. The second effect is the vasoconstricting effect, demonstrated in tumors, and confirmed on normal and tumor vessels by intravital microscopy [57]. This effect, termed vascular lock, induces prolonged entrapment of the drug within the tumors, providing better action of BLM or CDDP [58]. However, it also prevents inflow of the drug into tumors, if given after the delivery of electric pulses.

Evidence for electric pulses and electrochemotherapy actions on tumors exists in histological, physiological and numerical models [56,58]. Furthermore, it was also observed and demonstrated in clinical cases, where electrochemotherapy was used for the treatment of bleeding tumors [59,60]. However, relative contributions of vascular disrupting and vasoconstricting action of electrochemotherapy in overall electrochemotherapy effectiveness still remain to be determined. Some clinicians emphasize its great importance, predominantly in well vascularized tumors. However, vascular disrupting effect is not observed on bigger blood vessels, such as major hepatic arteries and veins, allowing treatment of tumors in vicinity of them, being the advantage over radiofrequency ablation which is a thermal ablative technique ineffective along bigger blood vessels due to the heat sink effect. Safety and efficacy of electrochemotherapy was recently demonstrated in tumors that were close to bigger vessels and in treatment of liver metastasis located between the major hepatic vessels [22].

Last but not least, involvement of immune response of the organism after electrochemotherapy was demonstrated to be important. Evidently, due to the heterogeneity of tumor cells in the tumors, in relation to their orientation, their size, and uneven drug distribution in the tumors, not all cells in the tumors can be effectively eradicated by electrochemotherapy [61]. This is due to the fact that not all the cells can be electroporated and/or the drug is not available to all the cells in the tumor. Both reasons stem from the inhomogeneity of the tumor [62]. Therefore, similarly as with other physical methods, e.g. radiotherapy, the remaining fraction of cells, when sufficiently low, can be eradicated by the effective immune response of the organism. In relation to this, evidence that immune competence of the organism is needed for the complete eradication of the tumors after electrochemotherapy was provided; namely in immunodeficient organisms the curability rate of the tumors was significantly lower than in immunocompetent ones [61]. This is thought to be related to antigen shedding after electrochemotherapy from the destroyed cells, which would activate immune cells within the tumors. Some indications also point to the recruitment of antigen presenting cells and boosting of CD11c and CD11b cells after electrochemotherapy of melanoma [63,64].

It needs to be stressed however, that the effect of electrochemotherapy is local; limited to the treated nodules, as the neighboring non-treated nodules do not respond. To gain systemic component some attempts were made to boost the immune response by cytokines (IL-2, IL-12, IL-15, GM-CSF, TNFα) providing some preclinical and clinical data on the possibility of combining electrochemotherapy and immunotherapies, to provide systemic antitumor activity [49,65-69].

Based on the mechanisms of action of electrochemotherapy, antitumor activity should be observed on all types of tumors tested, regardless of their histological origin. In fact electrochemotherapy is effective on all tumor types, but the effectiveness seems to vary somewhat, depending on the tumor type [50]. Also, recent review of clinical data provided evidence of different level of effectiveness of electrochemotherapy in different tumor types [20]. Although the underlying mechanisms are still to be fully determined, several mechanisms were already proposed. The first could be that there is intrinsic variability in tumor cell sensitivity to the drugs [50,68,70], the second that successful membrane permeability to some degree depends on tumor type although being sufficiently high for cell permeabilization [20,50,71], the third, that drug distribution and thus its availability in different tumors varies depending on the vascularization of the tumors [58], and the fourth, that the immunogenicity of the tumors is also involved [68].

Recently an attempt was made to address bioavailability of drug particularly in larger tumors by adding intratumoral delivery of drug in addition to systemic, i.e. intravenous drug delivery. Based on these results it seems that the variability in response due to pharmacological variances in drug distribution particularly in larger tumors could be circumvented by intratumoral drug administration [72-74]. Furthermore, the pharmacological *in vivo* data in patients need to be obtained in order to verify the drug dosage in the tumors that is required for effective electrochemotherapy. The drug dosage needed for treatment of smaller tumors may be lower than for larger ones (more than 2 cm in diameter), as well as for well vascularized tumors, like hepatocellular carcinoma, vs. metastases of colorectal carcinoma in liver that are less vascularized. Such studies would also clarify the therapeutic window, namely, is there really only 20 minutes time for the therapy [75] or the time could be longer; the current recommendations are based on observations of only a single patient. Gathering such pharmacokinetic and pharmacodynamics data would thus be of great clinical significance.

### Clinical data on electrochemotherapy

#### Formation of standard operating procedures

The initial clinical studies that were reporting on electrochemotherapy with BLM or CDDP, even though using somewhat different protocols, provided sufficient evidence for safe and effective use of electrochemotherapy, both in human and veterinary oncology [70,76,77]. Another milestone was achieved with preparation of the standard operating procedures (SOP) for electrochemotherapy using the Cliniporator device that were prepared in 2006 during the ESOPE project (European Standard Operating Procedures of Electrochemotherapy) [78]. The SOP were prepared based on the experience of the leading European cancer centers on electrochemotherapy [55]. The aim of this document was to define guidelines for safe and effective use of electrochemotherapy for treatment of cutaneous and subcutaneous tumors, when using the newly developed electric pulse generator Cliniporator. In particular, the SOP was aimed at oncologists that have no or little experience performing electrochemotherapy, and standardizing the therapeutic procedure. The treatment procedures were described in details, to the point of all necessary materials, and electrode selection for treatment, in specific clinical situation.

**Figure 1:**
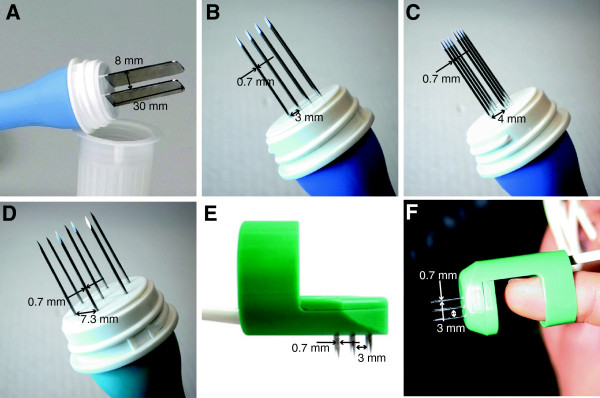
**Fixed geometry electrodes available from IGEA Srl. A)** Plate electrodes – recommended voltage 960 V. **B)** Linear needle electrodes – recommended voltage 400 V. **C)** Linear needle electrodes, front view. **D)** Hexagonal needle electrodes – recommended voltage 730 V. **E)** Finger electrodes with perpendicular needles; distance between rows is 4 mm – recommended voltage 400 V. **F)** Finger electrodes with axial needles – recommended voltage 400 V.

Briefly, in the SOP the decision tree was designed and was to be followed helping clinicians in decisions how to treat the patient, according to the number, size and thickness/depth of nodules to be treated. The limit for recommendation of intratumoral drug administration was set at the point of 5–7 nodules and tumor nodules up to 2 cm in diameter. However, on multiple nodules and tumor nodules larger than 0.8 cm, an intravenous drug administration was recommended. Furthermore, the choice of electrodes was suggested based on the size and the thickness/depth of the tumor nodule. The superficial and small (up to 1 cm) nodules were recommended to be treated by plate or needle row electrodes, whereas larger nodules were to be treated by hexagonal needle electrodes [55,78] (Figure 1). The procedure can be performed both in local or general anesthesia, which was left to the choice of the treating surgeon, but few and smaller nodules were recommended to be treated in local, while others in general anesthesia. The SOP also suggested that electrochemotherapy could be repeated in consecutive sessions.

**Figure 2:**
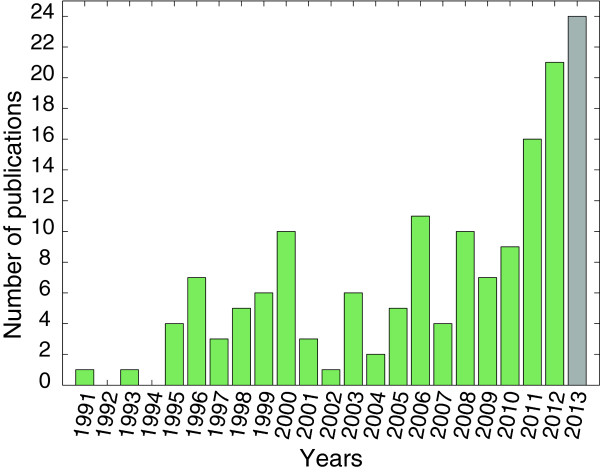
Number of publications on clinical electrochemotherapy over the years.

The preparation of the SOP was an important step which enabled the spread of the technology throughout Europe, providing the pulse generator certified for use in patients, standardized electrodes, and simple guidelines for medical doctors on how to use electrochemotherapy in treatment of superficial tumors [55]. Electrochemotherapy is being introduced into standard clinical practice in Europe and SOP undoubtedly facilitated its increasing usage in clinics [79]. The number of ongoing clinical trials (currently there are 6 ongoing studies in clinicaltrials.gov, and 5 in clinicaltrialsregister.eu, however, 3 entries are present in both databases) and the number of published studies is increasing (Figure 2), confirming the relevance of electrochemotherapy in cancer patient’s care.

Since the technology (electrochemotherapy) has spread throughout Europe, vast experience on its clinical applications is being gathered. It has expanded to the point that electrochemotherapy is now being used in different clinical situations, which were not predicted in the originally published SOP. Therefore, new SOP for cutaneous and subcutaneous tumors and eventually for deep-seated tumors are needed, in order to assure the effectiveness of the treatment and even more importantly the safety for the patients.

### Implementation of electrochemotherapy into standard of care: focus on cutaneous and subcutaneous tumors

New treatments provide benefit to the patients but at the same time they can put them at some new risk. Electrochemotherapy, in addition to already established skin directed therapies, like surgery, radiotherapy, topical and intralesional therapies, and other ablative techniques, offers itself due to its relative ease being performed on an outpatient basis as well as due to its safety and low toxicity. The feasibility and safety of electrochemotherapy was evaluated in the first few clinical studies on electrochemotherapy [2,80,81]. Due to good safety and toxicity profile, and only minor side effects, it was concluded that electrochemotherapy on superficial tumors is a safe treatment [19,78,82,83]. No serious effects related to electrochemotherapy have ever been reported. However, some minor immediate effect are observed, like minor irritation and uncomfortable sensation or pain associated with contraction of muscles in the vicinity of the electrodes that immediately subside after delivery of each electric pulse if performed in local anesthesia [78,84-86]. In addition, some late effects can also occur, like slight erythema, edema and sometimes necrosis of the tumor. All these effects are local, transient, minimal and well tolerated by patients; therefore electrochemotherapy can be performed in an outpatient setting. However, in some latest studies on locally advanced and metastatic soft tissue sarcomas [87] and large cutaneous recurrences of breast cancer [88], they observed longer-lasting periods of pain after electrochemotherapy treatment.

Beside good safety profile, electrochemotherapy on superficial tumors proved to be effective local tumor treatment. The effectiveness of single-session electrochemotherapy was recently extensively addressed in systematic review including meta-analysis [20]. After more than two decades of clinical application of electrochemotherapy, there was an indispensable need to consolidate current experience on electrochemotherapy from the effectiveness point of view and to establish the actual overall effectiveness of electrochemotherapy derived from data reported in clinical studies to-date. The responses of 1894 tumors from 44 eligible clinical studies published until October 2011 were included in the evaluation. The data demonstrated that the effectiveness of single-session electrochemotherapy on cutaneous and subcutaneous tumors is 59.4% for complete response (CR) and 84.1% for objective response (OR). Furthermore, outcomes of studies published before and after publication of the SOP were compared. Based on results obtained, we could presume that SOP improved outcome of electrochemotherapy (see Table 2). Partial and objective rates significantly increased, whereas non-response rate significantly decreased. However, CR rate remains almost the same. We probably cannot ascribe this improvement in electrochemotherapy outcome solely to the SOP, as other factors like experience gained by clinicians, as well as their training, and exchange of the experience, definitely contributed their share. Recently, an increasing number of new clinical studies on electrochemotherapy have been published, after the publication of the meta-analysis review. Therefore, we updated results of systematic review by including outcomes of 472 tumors from 16 additional studies published between October 2011 and August 2013 (Table 2). These results confirm positive influence of SOP on effectiveness of electrochemotherapy on cutaneous and subcutaneous tumors.

**Table 2:** Response rate of the tumors treated by electrochemotherapy, pooled from individual studies

**Publication period**	**No. of studies**	**No. of patients**	**No. of tumors**	**OR (%)**	**CR (%)**	**PR (%)**	**NR (%)**
**Before SOP**	19	175	592	458 (77.4%)^a, b^	362 (61.1%)	96 (16.3%)^c, d^	134 (22.6%)^e, f^
**ESOPE**	1	41	171	145 (84.8%)	126 (73.7%)	19 (11.1%)	26 (15.2%)
**After SOP (Oct 2011)**	25	294	1192	1047 (87.8%)^a^	712 (59.7%)	335 (28.1%)^c^	145 (12.2%)^e^
**After SOP (Aug 2013)**	41	519	1664	1478 (88.8%)^b^	1031 (62.0%)	447 (26.9%)^d^	186 (11.2%)^f^

The studies are grouped in those before, and after the ESOPE study, when the standard operating procedures were published, in 2006. OR = objective response (complete response + partial response); CR = complete response; PR = partial response; NR = no response (no change + progressive disease); ^a, b, c, d, e, f^ = statistically significant difference (*p* < 0.001, Chi-square test).

In addition to overall effectiveness of electrochemotherapy, the systematic review [20] addressed also differences in effectiveness of electrochemotherapy of cutaneous and subcutaneous tumors in clinical setting due to heterogeneous treatment conditions (i.e. chemotherapeutic drug, route of drug administration, and tumor type). Equal effectiveness of electrochemotherapy was demonstrated for BLM and CDDP administered intratumorally. However, significantly higher effectiveness for intratumoral than for intravenous administration of BLM was established. The results of electrochemotherapy after the intratumoral or intravenous drug administration basically cannot be compared. Due to the obvious difference in the drug concentrations in the tumors; after intratumoral administration higher drug concentration is delivered to the tumor than after intravenous administration. Currently most of the studies report clinical data after intravenous administration, so there may be imbalance between the two groups that are being compared. Furthermore, the differences in the response rate may be also due to the difference in the tumor size treated; tumors that were treated by intravenous route may have been larger, since the SOP was designed to utilize intravenous route for such situations. Recently it has been suggested that treatment of larger tumors with combined intravenous and intratumoral drug administration can be used with good success [73]. Future studies should be designed to evaluate the intratumoral route for larger tumors. Previous clinical [82] and preclinical [49] studies demonstrated the potential success of such approach.

It has been demonstrated that effectiveness of electrochemotherapy varies with tumor type [20]. Use of often repeated statement about equal effectiveness of electrochemotherapy, regardless of tumor type based on available clinical results appears to be unjustified. Among all types of tumors, the highest effectiveness of electrochemotherapy was achieved on basal cell carcinoma and the lowest on squamous cell carcinoma tumors, presumably because squamous cell carcinoma tumors were usually larger in size than basal cell carcinoma tumors, and also reflecting the fact that basal cell carcinomas tend to recur and metastasize at a lower rate than all other skin tumors.

Currently, there are several registries that pool the data on patients treated with electrochemotherapy; the INSPECT and at least two Italian registries [89]. Some of them gather data on specific tumor type, melanoma, head and neck tumors, and the other (INSPECT) on any kind of tumor histology. These data, if appropriately collected, will help development of electrochemotherapy, and indicate whether drug dosage, route of administration, or electric pulse treatment needs to be adjusted for higher effectiveness of electrochemotherapy. Currently all the clinical data report on tumor response, however in future studies the data on patients response are needed in order to indicate the effect of electrochemotherapy on tumor progression free interval or overall survival. Only then another meta-analysis can unambiguously demonstrate the differences in the response rate of different tumor types, based on well synchronized data collection, and sufficiently long follow-up of patients [90].

Although skin tumors are primarily treated by surgery and radiotherapy, cutaneous metastases are not rare. Furthermore, improved treatment of metastatic cancer results in longer life expectancy, which also results in increased incidence of skin metastases. Skin metastases can represent important problems for patients and physicians – bleeding metastasis, local infections, pain, and the consequences of radical treatment by surgery may lead to disfigurement and greatly impaired quality of life. The treatment of skin metastases is thus becoming increasingly important. Not just improve in patients’ quality of life but also long-lasting local disease control of cutaneous metastases treated with electrochemotherapy was confirmed in some clinical studies [73,91,92].

The current prevalent use for electrochemotherapy in skin tumors is focused on palliation in patients with numerous skin metastases, or patients who have undergone many previous treatments. This use is the most documented and investigated, and is also most supported, e.g. by the NICE guidelines, as we detail in the following section. Electrochemotherapy has also been used in less aggressive non-melanoma types of skin cancer for treatment of primary tumors, such as basal cell carcinoma. In such cases, treatment needs to be very efficient to be considered for use in comparison with surgery or radiotherapy. The main use of electrochemotherapy in such cases would be in areas where there is too little tissue to be able to perform radical resections or where surgery outcome could lead to greatly diminished patients’ quality of life, or where a good cosmetic outcome is desired, such as, the head and neck area [93], the cheeks [94,95], the nose [82,96] or near the eyes [97].

In order to get wide acceptance of any kind of treatment, it has to be included in (national) guidelines for a specific treatment, authorized by national or international body, and then it will also enter into the national health system and become eligible for reimbursement. The acceptance is when the effectiveness of the new therapy is evidence based, compared to the other ablative techniques that are currently used for cutaneous tumors/metastases. Therefore only comparative studies will unambiguously demonstrate its effectiveness. Another issue is the acceptance by the patients, morbidity and hospitalization, where electrochemotherapy may have its advantages over other ablative techniques.

Currently, electrochemotherapy clinical application is mostly limited to Europe and is routinely applied in everyday clinical practice in 130 clinical centers (information obtained from IGEA SpA upon request in January 2014). Accounting for differences among EU countries, electrochemotherapy iscurrently reimbursed by the national health insurances in Switzerland, Austria, Germany, Denmark, Spain, UK, Italy, Portugal, and Slovenia. Reimbursement activities are ongoing in Poland and France (data obtained from IGEA Srl in January 2014). In most of these countries it has been acknowledged in their treatment guidelines, as well as recommended in European for the treatment of melanoma [98]. However, except for melanoma, electrochemotherapy still awaits recognition in guidelines for treatment of other cancers. The exception is NICE guidelines, where besides melanoma, electrochemotherapy was recognized as treatment option also for the treatment of primary basal cell carcinoma and squamous cell carcinoma [99], and in German guidelines provided by Dermatologic society [100]. Further advantage of electrochemotherapy is that it can be implemented in developing countries, where big facilities lake radiotherapy departments are not available, and the cost of systemic therapy is too high. In such environment, electrochemotherapy could be the option to help patients with cancer. A further step forward would be also use of non-chemotherapeutic drugs that do not require specific preparation or handling in oncology centers. The first attempt has already been made, the use of calcium, which has proved its activity in preclinical studies on electrochemotherapy (Table 1) [31], and the first clinical trial on calcium use in electrochemotherapy has been initiated (ClinicalTrials.govidentifier: NCT01941901). This approach would give electrochemotherapy advantage to be implemented outside the oncology centers, also in private practice of dermatology clinics.

Summarizing, electrochemotherapy is currently accepted for treatment of cutaneous and subcutaneous metastases and palliation of skin tumors, but lacks evidence for using electrochemotherapy as curative treatment of primary tumors. For this, additional studies need to be designed and coordinated, which will compare electrochemotherapy with current standard of care, like surgery and radiotherapy in the case of basal cell carcinoma.

### Current developments of electrochemotherapy

#### Visceral and deep-seated tumors

Electrochemotherapy is now being developed and evaluated for treatment of visceral or deep-seated tumors [22,23]. In order to do this, several steps need to be accomplished, including adjustment of the technology and clinical studies that will prove the safety and effectiveness of the treatment in phase I/II studies. The technology is already being modified for the treatment of tumors larger than 3 cm in diameter, since the current technology provides treatment of tumors smaller than 3 cm. The other adjustments are in the line of safety, predominantly in organs adjacent to the heart, where the delivered electric pulses can interfere with functioning of the heart. Since the heart is especially susceptible to induction of life-threatening arrhythmias if electrical pulses are delivered during the so-called vulnerable period of the ventricles which coincides with T wave in ECG, synchronization of pulses presents another engineering challenge. The vulnerable period can be avoided by synchronizing the delivery of the pulses with the QRS complex of the heartbeat [101-103]. This is an important consideration particularly for clinical trials that are on-going, i.e. for treatment of liver [22], bone [104] and brain metastases [105], as well as for the treatment of big breast chest wall recurrences [88,106].

Several characteristics make electrochemotherapy a potential candidate for treatment of visceral and deep-seated tumors. These are the ability to treat volumes with diameters in excess of 3 cm, which is a typical limit for thermal therapies, such as radiofrequency and microwave ablation; the advantage of organ sparing effect, and possibility of treatment in the vicinity of major blood vessels. Along with these benefits come additional engineering challenges, among them the requirement of complete tumor nodule coverage by sufficiently high electric field [51,52], that can be achieved by patient specific pre-treatment planning [107], the availability of suitable electrodes for delivery of electric pulses [108,109], synchronization of pulse delivery with ECG [102,103,110], and intraoperative guidance of electrode positioning.

The overview of the implementation of electrochemotherapy in treatment of deep-seated tumors demonstrates that new electrodes are being developed and used in treatment of various tumor types and locations (Table 3).

**Table 3:** Different types of electrodes developed for electrochemotherapy of visceral and deep-seated tumors

**Type of electrodes**	**Location and type of tumors treated**	**Institution performing treatment**	**References/Clinical trial number**
**Long needle (Figure**3**)**	Metastases of colorectal tumors in liver bone metastases soft tissue sarcomas	Institute of Oncology Ljubljana, Slovenia Istituto Ortopedico Rizzoli, Bologna, Italy	[22], NCT01264952
			[104]
			[87]
		Veneto Region Oncology Research Institute of Padova, Italy	
**Endoluminal (Figure**4**)**	Colorectal, gastric and esophageal tumors	Cork Cancer Research Center, Ireland	[108], NCT01172860
**Expandable “umbrella” type (Figure**5**)**	Brain tumors	Copenhagen University Hospital at Herlev, Denmark	[105], NCT01322100

**Figure 3:**
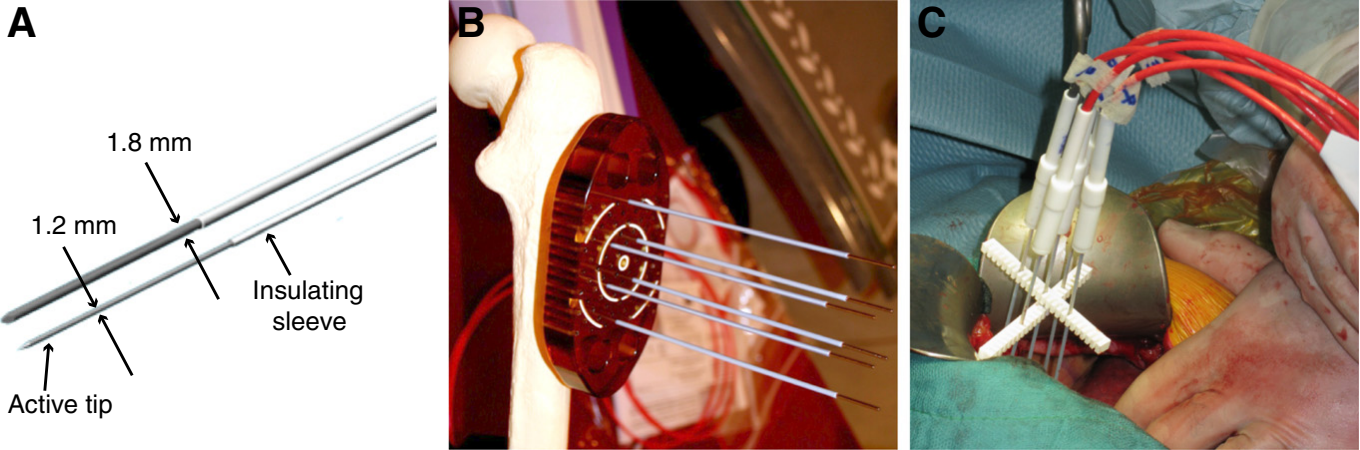
**Variable geometry needle electrodes available from IGEA Srl. A)** Two needle types are available. Active tip length is either 3 or 4 cm. **B)** 1.8 mm electrodes with trocar tip used for drilling into bone. Shown with a bone model. **C)** 1.2 mm electrodes used during open surgery to treat liver metastasis.

**Figure 4:**
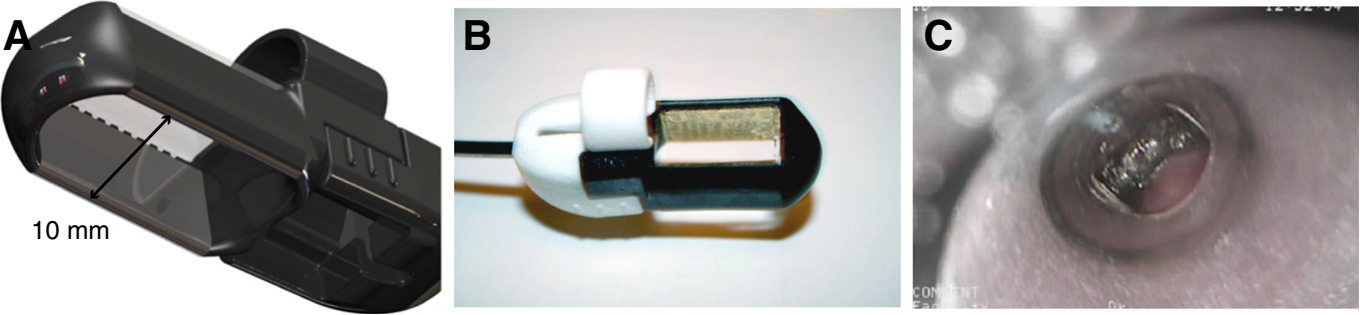
**Endoscopic electrode EndoVe. A)** CAD model. **B)** Photography of an actual sample – recommended voltage 1000 V. **C)** View of the target lesion during endoscopic procedure.

**Figure 5:**
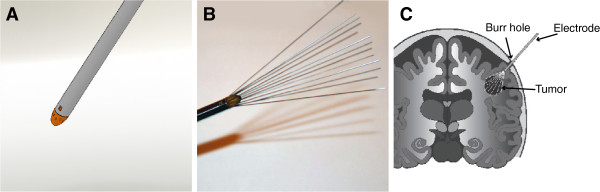
**Retractable brain electrodes. A)** Rendering of the electrode in fully retracted state. **B)** Electrode in fully extended state. Recommended voltage is 1000 V for the fully extended electrodes. **C)** Diagram of the insertion into brain [105].

Basically three types of electrodes have been developed and are being used:

• The first ones are long needle electrodes that are inserted into the tumor and surrounding tissue in order to safely cover the tumor and achieve an appropriate margin (Figure 3). Such electrodes are being tested for treatment of liver metastases of colorectal tumors, where safety and effectiveness have been reported [22]. Such technology can be implemented also for treatment of other types of tumors, such as hepatocellular carcinoma or cholangiocarcinoma. This long needle technology is being tested also for treatment of soft tissue sarcomas [87] and for bone metastases [104], based on solid preclinical evidence [111]. However it could be implemented also for other large (more than 3 cm) tumors located deeper under the skin, like in squamous cell head and neck tumors.

• The second are endoluminal electrodes that are being tested for the treatment of colorectal cancer but could be used also for the treatment of esophageal tumors (Figure 4). The ongoing clinical trial in treatment of recurrent colorectal tumors has included so far just a few patients, but has proven feasibility of such approach with good antitumor effectiveness [112].

• The third are the electrodes that are aimed at treatment of brain tumors [105,109] (Figure 5). The electrodes have proven to be suitable for the treatment of the brain tumors, through the skull. Unfortunately due to slow recruitment the trial was stopped, but a new one is being prepared (*personal communication*). Such electrodes could potentially be used also for treatment of liver tumors, similarly as percutaneous radiofrequency ablation, with the development of appropriate monitoring system of electrode guidance into the tumors.

### Conclusion and remaining challenges

A new treatment or medical technology can be adopted in the clinics after it has proven to be safe and effective. This is the prerequisite, but not sufficient step. It needs to be established as a practical and safe use among many indications for treatment of cancer at various stages of the disease. Electrochemotherapy is now at the stage of palliative treatment in most indications, or in the case other treatments have failed. Therefore, controlled studies will probably not suffice to bring electrochemotherapy along the established ablative techniques (e.g. radiofrequency ablation) or surgery – controlled, randomized clinical trials are needed, but will be difficult to execute. The other option, based on the current evidence, is that controlled individualized trials will be launched, extending the treatment indications for also less advanced stages of the disease. In the current situation it is of utmost importance that no serious adverse events are reported, since this may shed uncertainty on its development. Consistent results regarding effectiveness after use by clinicians who are not original developers is also a critical step in the growth of a new treatment.

Electrochemotherapy is in its early phase of clinical acceptance, currently the focus being on skin tumors and metastasis. Further work is however required. In treating skin tumors and metastasis electrochemotherapy needs to be compared against current standard treatments; e.g. for basal cell carcinoma it needs to be compared to radiotherapy and/or surgery. This will also require a five-year follow-up!

In general the body of data needs to be collected in multicenter prospective randomized controlled studies in which response per patient rather than per tumor nodule is observed. Additionally, focus will need to be on tumor recurrence free interval, and since electrochemotherapy is a local treatment, quality of life improvement should probably be one of most important outcomes, rather than patients’ lifespan. Some of these data will also become available from clinical data registries like InspECT, IMI and GIDO.

The preparation of new/updated Standard Operating Procedures (SOP) will undoubtedly have to deal with differences in responses between differently sized tumors and different types of tumors. Original SOP and decision tree regarding the number of nodules and route of drug administration (intratumoral or intravenous) were based on the experience of the developers and not based on controlled studies determining appropriate dose or administration route. While effective and facilitating the advancement of this treatment modality, further refinement of SOP will be needed to get full acceptance from the medical community. Data on clinical pharmacokinetics and pharmacodynamics of bleomycin and cisplatin in electrochemotherapy would also be important to obtain. Among the studies still needed are dose response evaluations for both intravenous and intratumoral routes. This may be necessary for several indications particularly relating to size, location and type of tumor. In a properly designed study different responses to electrochemotherapy with respect to the tumor size could be addressed; as well as the need for combination of intravenous and intratumoral drug delivery as suggested for efficient treatment of large tumors.

The other challenge would be also to reduce intensity or extent of muscle contractions in order to eliminate the use of muscle relaxants and consequently also pain during electrochemotherapy. The first efforts in this direction have been done by suggesting application of high-frequency bipolar electroporation pulses [113].

The remaining challenge is also to address specificity and sensitivity of electrochemotherapy treatment and to determine its therapeutic index. It had not been systematically addressed before how does healthy tissue respond to electrochemotherapy.

In addition, electrochemotherapy can be implemented in combination with surgery as neoadjuvant treatment. Specifically, with such approach tumors located in surgically difficult to reach positions, due to the involvement of some critical physiological structures (e.g. nerves and blood vessels) or due to potential damage on organs and functions (e.g. sight, hearing, speech, eating) can be treated in order to reduce tumor burden before surgical removal. This approach has already been demonstrated to be useful and effective [95,114-118]. The downsizing of the tumors can facilitate the surgical intervention, with long term tumor control, and organ sparing effect. The other approach is that electrochemotherapy would be used after surgical removal of the bulk of the tumor, and electrochemotherapy would be used to treat the remaining tumor mass and achieve appropriate margins. There is no report on such approach yet, however it has been successfully used in treatment of sarcoids in horses (*personal communication, no reference is available*). Either of these approaches needs further developments, which only with time will provide enough evidence to implement them into a broader clinical practice.

Electrochemotherapy could be used also as concomitant treatment with other treatment modalities. One of the well explored applications is the use of electrochemotherapy as radiosensitizing approach. BLM and CDDP are well known radiosensitizers. Therefore, electrochemotherapy could, by increased intratumoral accumulation, potentiate radiation response of tumors without normal tissue damage. This was proven in animal models in several reports, with CDDP and BLM, in single and fractionated irradiation regimen [50,119,120]. However there are only a few clinical reports following this idea [121-123].

The other treatment combinations would be in combination with targeted drugs, and immunostimulatory approaches, either in the form of biologic therapy or in the form of gene therapy. Some attempts have already been made, predominantly in combination of electrochemotherapy with gene electrotransfer on preclinical level [49,66-69], however the approach needs clinical verification in clinical trials. It is presumed that such combined therapy can add a systemic component, by boosting the specific immune response to tumors that was elicited by antigen shedding from tumors after electrochemotherapy. However, there is no clinical evidence that after electrochemotherapy the non-treated nodules would respond.

In today’s economy additional health economics studies will have to be performed clearly showing acceptable price for the benefit that electrochemotherapy can bring to the patients [124]. Electrochemotherapy certainly has potential in this respect as the drugs currently used are inexpensive and there is the potential for the development of low cost instruments and electrodes.

Treating deep-seated tumors requires even more effort, time and resources. Even though the technology of image guided transcutaneous insertion of electrodes is available [125], pretreatment planning development for electrochemotherapy is still in its early development [126]. Currently electric field distribution prediction is calculated in 3D models using finite element methods numerical modeling, taking into account different electric properties and increase in conductivity due to electroporation. Optimization of placement of electrodes is thus performed based on criteria function which for electrochemotherapy requires tumor tissue to be covered with sufficiently high electric field [127]. Uncertainties in tissue conductivity and exact positioning of electrodes relative to the target tissue and each other can however greatly affect tumor treatment outcome [128]. In addition, cell kill based on electrochemotherapy model still needs to be developed in order to be able to predict tumor treatment outcome. Any approach, which is not covered by the SOP, can benefit greatly from treatment planning. Although conclusive proof of treatment planning’s benefit has not yet been established through controlled studies, there are several indications, that it is beneficial. At the very least, it can serve as tool for scaling from smaller to larger tumors, since the scaling is non-linear due to electroporation’s effect on tissue electric properties. Algorithms designed for treatment planning can also be used in novel electrode designs [109].

Achieving acceptance of a new clinical treatment for cancer is a huge project that takes considerable time, orchestrated efforts and a lot of resources, whereas biomedical engineers tend to look at medical progress in the short term. Appropriately controlled studies that take all parameters into consideration are a critical part of bringing a new approach to broad acceptance. Focusing on specific cancer targets to gain initial acceptance will open up additional treatment options and facilitate acceptance of the therapy. Apart from x-ray all other techniques and treatments needed 20 years or so to gain its place. Needless to say the route is not always straightforward, nor is it always successful. The regulations in Europe and USA are quite different, and specific, and costly. However, overall there are no big differences between the two markets [129,130]. There are new large markets that will affect usual strategies that companies have in bringing new technologies and treatments to the patient.

## Competing interests

DM holds patents on electrochemotherapy that are licensed to and is occasionally consulting IGEA S.r.l, Italy – a producer of device and equipment for electrochemotherapy. The other authors declare no competing interests.

## Authors’ contributions

DM conceived the review, and participated in its design and coordination and helped to draft the manuscript. BM made the systematic review of newly published clinical papers and wrote parts of the manuscript and corresponding figures and tables. BK made the electrode figures and wrote parts of the manuscript. RH helped draft the manuscript and participated with critical information to achieve a comprehensive review and evaluation. GS wrote parts of the manuscript and supervised the preclinical and clinical parts. All authors read and approved the final version of the manuscript.
